# Assessment of Transmission in Trachoma Programs over Time Suggests No Short-Term Loss of Immunity

**DOI:** 10.1371/journal.pntd.0002303

**Published:** 2013-07-11

**Authors:** Fengchen Liu, Travis C. Porco, Kathryn J. Ray, Robin L. Bailey, Harran Mkocha, Beatriz Muñoz, Thomas C. Quinn, Thomas M. Lietman, Sheila K. West

**Affiliations:** 1 F.I. Proctor Foundation, University of California, San Francisco, California, United States of America; 2 Department of Ophthalmology, University of California, San Francisco, California, United States of America; 3 Department of Epidemiology and Biostatistics, University of California, San Francisco, California, United States of America; 4 Faculty of Infectious and Tropical Diseases, Clinical Research Department, London School of Hygiene and Tropical Medicine, London, United Kingdom; 5 Kongwa Trachoma Project, Kongwa, Tanzania; 6 Wilmer Eye Institute, Johns Hopkins Hospital, Baltimore, Maryland, United States of America; 7 Johns Hopkins Center for Global Health, Johns Hopkins University, Baltimore, Maryland, United States of America; University of California San Diego School of Medicine, United States of America

## Abstract

Trachoma programs have dramatically reduced the prevalence of the ocular chlamydia that cause the disease. Some have hypothesized that immunity to the infection may be reduced because of program success in reducing the incidence of infection, and transmission may then increase. Longitudinal studies of multiple communities would be necessary to test this hypothesis. Here, we quantify transmission using an estimated basic reproduction number based on 32 communities during the first, second, and third years of an antibiotic treatment program. We found that there is little to no increase in the basic reproduction number over time. The estimated linear trend in the basic reproduction number, 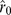, was found to be −0.025 per year, 95% CI −0.167 to 0.117 per year. We are unable to find evidence supporting any loss of immunity over the course of a 3-year program. This is encouraging, as it allows the possibility that repeated mass antibiotic distributions may eliminate infection from even the most severely affected areas.

## Author Summary

Trachoma, caused by repeated infections by the ocular strains of *Chlamydia trachomatis*, is the most common infectious cause of blindness in the world. Treatment for trachoma includes mass azithromycin treatments to the entire community. To reduce the prevalence of infection, the World Health Organization (WHO) advocates at least three annual community-wide distributions of oral antibiotics in affected areas, with further mass treatments based on the prevalence of trachoma. Trachoma programs have dramatically reduced the community prevalence of infection, and some have argued that lowered prevalence of infection may lead to reductions in immunity, and that less immunity may in turn lead to increased transmission from what infection remains. Here, we used a stochastic transmission model to analyze data collected from a 3-year antibiotic treatment program (a 32-community, cluster-randomized clinical trial in Tanzania) to assess whether or not transmission actually increases during elimination campaigns. We found no evidence supporting any increase in transmission over the course of the program. The absence of a short term increase in transmission as the prevalence decreases is good news for trachoma programs.

## Introduction

The World Health Organization has targeted trachoma for elimination by the year 2020 [1]. Repeated mass oral azithromycin distributions have been a cornerstone of the treatment strategy. Theoretically, repeated treatments may eventually eliminate infection from even the most severely affected areas [2], [3]. In practice, distributions have dramatically reduced the prevalence of infection in a number of locations [3], [4], [5], [6], [7], [8], [9]. However, there remains concern that resistance may develop or that loss of immunity may prevent complete elimination [10], [11], [12], [13], [14]. While no stable chlamydial drug resistance has yet been observed, a loss of immunity is possible. Individuals who had not been exposed to infection recently might have less protection than they had had when the infection was more prevalent. An increase in transmission during the course of a program could indicate loss of immunity. Multiple communities would need to be monitored, to assess whether random fluctuations may explain observed differences over time. Here, we analyzed multiple communities from the Tanzanian portion of the Program for the Rapid Elimination of Trachoma trial (PRET [15]) using a stochastic model of transmission, to assess the initial reproductive number for transmission over time.

## Methods

### Clinical and laboratory results

Communities were monitored as part of a cluster-randomized, trachoma treatment trial in Tanzania [15], [16]. In brief, 32 communities in Tanzania were randomized in a two by two factorial design. The first factor was the use of standard versus enhanced coverage with annual mass antibiotic treatment; the second factor was the use or disuse of a rule whereby mass antibiotic administration would be discontinued based on ongoing monitoring. In fact, the use of this rule never led to discontinuation of mass antibiotic administration during the first three years. Thus, all 32 communities received treatment at baseline, 12, and 24 months. A baseline census was conducted in all 32 communities, and again at 12, 24, and 36 months. One hundred randomly selected children aged 0–5 years were examined at baseline, and at 6, 12, 18, 24, 30, and 36 months post baseline. A dacron swab was passed 3 times over their inverted right upper conjunctiva, and processed for the presence of chlamydial DNA as previously described [15]. Our stochastic transmission model was fit to the estimated prevalence of infection at 6, 12, 18, 24, 30, and 36 months.

### Ethics statement

The study received ethical approval from institutional review board (IRB) of the Johns Hopkins University School of Medicine, the University of California San Francisco, and the Tanzanian National Institute for Medical Research, and was carried out in accordance with the Declaration of Helsinki. All subjects provided informed consent. The informed consent given was oral, because 1) verbal consent is the most ethical way to obtain consent, due to the high illiteracy rates in the study area, 2) IRB approved the use of the oral consent procedure for this study, 3) this oral consent is documented on the registration form for each study participant prior to examination in the field.

### Modeling methods

We constructed a stochastic transmission model of transmission of *Chlamydia trachomatis* infection over time [17], [18], [19], [20]. For community *j* (*j* = 1,…,32), we assumed a population of size *N_j_*, taken from the number of pre-school children found in the census at the time of treatment (baseline, 12 months, or 24 months). We assumed a classical SIS (susceptible-infective-susceptible) model structure, assuming that the force of infection is proportional to the prevalence of infection in the population with proportionality constant *β*, and a constant per-capita recovery rate *γ* (month^−1^). Between periods of treatment, we assumed that the probability *p_i_*(*t*) that there are *i* infectives in the population obeyed the following equations:
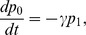


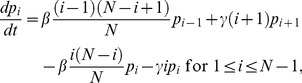
(1)and
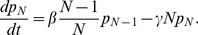
These equations were applied to each village *j*, though we have suppressed a subscript *j* for clarity in Equation (1).

### Statistical methods

To estimate the transmission coefficient, we used data collected six months and twelve months after each treatment. The model was fit to each of three years. For comparing transmission rates, we initialized the model with observations taken six months after treatment, and we estimated the transmission parameter based on values observed six months after that. Thus, we modeled the time periods from 6 to 12 months, 18 to 24 months, and 30 to 36 months.

For each year, initial values for *p_i_*(*t*) were determined from as follows. From a population of size *N_j_* of which the number *Y* of infectives equals *i*, the probability 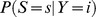 that *s* positives are observed from a sample of size *M_j_* are sampled is given by the hypergeometric distribution: 
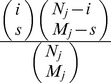
. We assumed a beta-binomial prior 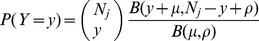 (where the shape parameters 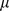 and 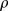 were computed from the observed distribution of infection of 32 villages at 6 months, 18 months and 30 months, and *B*(*x,y*) is the beta function [21])) for all values of *y*. Application of Bayes' theorem yields
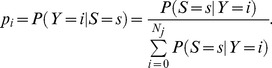
(2)For each community *j*, we used the most recent census data to determine the community size *N_j_*. The initial condition was determined from Equation (2), and the system numerically integrated for six months. Let *S_j_* be the number of positive individuals detected in the sample at the end of the period (for community *j*). Given the number *i* of infected individuals, we computed the probability of the observed data according to 
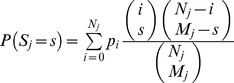
 (where *M_j_* here denotes the sample size at the end of the period). We assumed independent communities, and thus we might maximize the sum of the logarithm of the above expressions (summing over all communities).

We assumed specific values of *γ* (see Table 1) and estimated the value of *β* that maximizes the total loglikelihood given 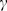. Standard errors were obtained from the observed Fisher information. We estimated the change, *m* (year^−1^), in the transmission coefficient per year by finding maximum likelihood estimates of *β*_1_ and *m* (with *β*_2_ = *β*_1_+*m*, *β*_3_ = *β*_1_+2*m*). For statistical comparison, we also used all time periods to estimate a single (constant) transmission coefficient for all three periods. The basic reproduction number is given by 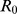 = *β/γ*; thus, estimated values of 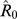 may be computed by dividing the estimated transmission coefficient by *γ*. The estimated annual change in the basic reproduction number, 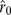, may be computed by 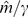.

**Table 1:** Estimated initial reproductive number and the change per year.

Scenario	Mean duration of infection (1/*γ*)	Estimated 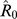 (95% CI)				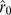 (95% CI)*
		Overall	1^st^ year	2^nd^ year	3^rd^ year	
Base Case	6-month	1.39 (1.28, 1.49)	1.40 (1.26, 1.55)	1.38 (1.09, 1.67)	1.35 (0.92, 1.78)	−0.025 (−0.167, 0.117)
Sensitivity Analyses	18-month	1.90 (1.62, 2.19)	11.87 (1.48, 2.26)	1.92 (1.15, 2.70)	1.96 (0.82, 3.13)	0.054 (−0.326, 0.434)
	12-month	1.64 (1.45, 1.84)	1.64 (1.37, 1.91)	1.65 (1.12, 2.18)	1.66 (0.87, 2.45)	0.012 (−0.248, 0.273)
	3-month	1.26 (1.19, 1.31)	1.28 (1.20, 1.37)	1.24 (1.08, 1.41)	1.20 (0.96, 1.45)	−0.041 (−0.122, 0.039)

* : the 95% CI of 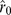 was obtained from the observed Fisher information.

We also estimated an alternative model in which instead of varying the transmission coefficient over time, we instead assumed a constant transmission coefficient, and instead modeled the recovery rate in year *i* (*i* = 1,2,3) according to 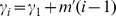, where 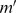 (month^−1^ year^−1^) is the annual change in recovery rate.

Previous models have estimated the duration of infection 1/*γ* to be from 3 months to 12 months [17], [19], [20]. As a base case, we assumed a mean duration 1/*γ* of 6 months; as a sensitivity analysis, we varied the mean duration from 3 to 18 months.

All calculations were performed using *R* (version 2.14.1, *R* Foundation for Statistical Computing, Vienna, Austria).

## Results

The numbers of 0–5 year-old children tested for the presence of ocular chlamydia were 3199 (baseline), 3198 (month 6), 3191 (month 12), 3200 (month 18), 3199 (month 24), 3194 (month 30), and 3153 (month 36). The estimated prevalence of ocular chlamydial infection by PCR at baseline was 22.0% (standard deviation 10.1%), at 6 months 10.5% (SD 4.7%), at 12 months 13.0% (SD 6.4%), at 18 months 7.1% (SD 4.4%), at 24 months 8.6% (SD 7.3%), at 30 months 3.5% (SD 2.5%), and at 36 months 4.7% (SD 3.3%).

Assuming a mean duration of infection of six months with beta-binomial prior (choosing the shape parameters to match the observed mean and variance of all villages cross sectionally at each initialization time), we found the basic reproduction number to be 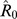 = 1.39 (95% CI: 1.28 to 1.49). For the first year, 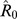 = 1.40 (95% CI: 1.26 to 1.55); for the second year, 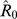 = 1.38 (95% CI: 1.09 to 1.67), and for the third year, 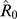 = 1.35 (95% CI: 0.92 to 1.78).

The estimated change per year in the reproductive number, 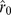, was found to be −0.025, 95% CI −0.167 to 0.117, see Table 1. Similar findings were obtained when we assumed other values for the mean duration of infection: three months, 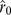 = −0.041 (95% CI: −0.122 to 0.039) year^−1^; twelve months, 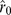 = 0.012 (95% CI: −0.248 to 0.273) year^−1^; eighteen months, 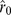 = 0.054 (95% CI: −0.326 to 0.434) year^−1^. Regardless of the assumed duration of infection, we find point estimates for the annual change in the basic reproduction number which are near zero. The confidence intervals are wider when a longer duration of infection is assumed, and these intervals include zero (i.e., no change) in every scenario.

Similar findings were obtained when a different choice of prior was used. Specifically, we assumed a uniform distribution as the prior distribution for the number of infected individuals; this yielded an overall 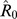 = 1.30 (95% CI: 1.19 to 1.41) based on a pooled estimate assuming a constant *β* for all three years (i.e. assuming *m* = 0) and a mean duration of infection of six months. The corresponding estimate for the estimated change per year in the reproductive number is 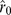 = −0.084 (95% CI: −0.227 to 0.058) year^−1^. Choosing other values of the mean duration together with the uniform prior similarly yielded the following results: three months, 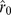 = −0.072 (95% CI: −0.152 to 0.009) year^−1^; twelve months, 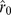 = −0.103 (95% CI: −0.366 to 0.161) year^−1^; eighteen months, 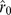 = −0.118 (95% CI: −0.502 to 0.266) year^−1^.

We estimated the change in the recovery rate *γ*, assuming a constant transmission coefficient (the optimal value when infection duration was assumed to be 6 months). The estimated linear trend in recovery rate was found to be 0.013 (95% CI: −0.009 to 0.035) month^−1^ year^−1^, with the estimated recovery rate in the first year given by 0.177 (95% CI: 0.154 to 0.20) month^−1^ year^−1^. This model yields a substantially similar interpretation as the previous model.

## Discussion

Using a transmission model and data collected from a 32-community, cluster-randomized clinical trial in Tanzania, we found no evidence of increased transmission from the 1^st^ through the 3^rd^ year of treatment. In fact, our estimates of the reproduction number of the infection were very similar for each year, suggesting no loss of immunity.

Others have proposed an arrested immunity hypothesis, in which the development of protective immune responses is decreased as the duration of chlamydial infection is decreased. It has been suggested that an increased incidence of infection in the presence of a decreased seroprevalence in Finland and British Columbia is due to this phenomenon [10], [22]. Trachoma programs have offered an ideal setting to test this hypothesis. A study in Vietnam suggested that a single community with more antibiotic treatment had more rapid return of infection than another less treated community, and that this may be due to a loss of immunity [11]. Without a larger number of treated communities, it was not possible to assess whether the magnitude of this paradoxical result would have been expected by chance alone [23]. In this present study, the large number of longitudinally monitored communities offered more power, yet we were unable to document any evidence of increased transmission with more treatment.

There are several reasons this analysis of these data might fail to detect an increase in transmission, even if such an increase in fact exists. Two years may not be long enough for immunity to wane, although the period in which an earlier study suggested waning was only one year [11]. Uncertainty in the average duration of infection is a potential source of model misspecification, although a sensitivity analysis suggests that the reproductive number and estimated change are not sensitive to the value of infection duration. Even though the trial from which these data came is one of the larger trachoma studies performed, 32 communities may not be a large enough sample size to detect a modest increase in transmission. It is possible that a loss of immunity did occur, but that any effect on transmission was balanced by a decrease in transmission due to other factors; other studies have reported that per-infectious case transmission may decrease with decreasing prevalence, perhaps due to a decrease in the diversity of strains at lower prevalence [19], [24]. Finally, we have assumed that transmission is proportional to the number of infectious cases and number of susceptible cases (mass action); if this is not the case, then this may have masked increased transmission at the later, lower prevalence [19].

Models have predicted that if transmission per infectious case remains constant, repeated distributions can eliminate infection from even the most severely affected communities [2], [3]. Longitudinal studies have confirmed that local elimination is possible [5], [6], [25], [26], [27]. However, these successes might not hold in the future, if antibiotic resistance were to develop, or if a loss of immunity resulted in increased transmission. The absence of a short term increase in transmission as the prevalence decreases is good news for trachoma programs.
