# Correction: Assessment of Transmission in Trachoma Programs over Time Suggests No Short-Term Loss of Immunity

**DOI:** 10.1371/annotation/c64f02de-c1f1-48ee-ac79-06b5a6c7b65a

**Published:** 2013-07-31

**Authors:** Fengchen Liu, Travis C. Porco, Kathryn J. Ray, Robin L. Bailey, Harran Mkocha, Beatriz Muñoz, Thomas C. Quinn, Thomas M. Lietman, Sheila K. West

There is a misprint in the data of Table 1, row 4, column 4 (Sensitivity Analyses and 1st Year, respectively). This data should read "1.87 (1.48, 2.26)."

